# Complex Electrical Conductivity of Biotite and Muscovite Micas at Elevated Temperatures: A Comparative Study

**DOI:** 10.3390/ma13163513

**Published:** 2020-08-09

**Authors:** Vassilios Saltas, Despoina Pentari, Filippos Vallianatos

**Affiliations:** 1 Institute of Physics of the Earth’s Interior and Geohazards, Hellenic Mediterranean University Research Center, 73100 Chania, Greece; fvallian@geol.uoa.gr; 2 Laboratory of Inorganic and Organic Geochemistry and Organic Petrography, Department of Mineral Resources Engineering, Technical University of Crete, 73100 Chania, Greece; pentari@mred.tuc.gr; 3 Department of Geophysics-Geothermics, Faculty of Geology and Geoenvironment, National and Kapodistrian University of Athens, 15772 Athens, Greece

**Keywords:** mica, biotite, muscovite, impedance spectroscopy, electrical conductivity, electric modulus, high temperature

## Abstract

The unique physicochemical, electrical, mechanical, and thermal properties of micas make them suitable for a wide range of industrial applications, and thus, the interest for these kind of hydrous aluminosilicate minerals is still persistent, not only from a practical but also from a scientific point of view. In the present work, complex impedance spectroscopy measurements were carried out in muscovite and biotite micas, perpendicular to their cleavage planes, over a broad range of frequencies (10^−2^ Hz to 10^6^ Hz) and temperatures (473–1173 K) that have not been measured so far. Different formalisms of data representation were used, namely, Cole-Cole plots of complex impedance, complex electrical conductivity and electric modulus to analyze the electrical behavior of micas and the electrical signatures of the dehydration/dehydroxylation processes. Our results suggest that ac-conductivity is affected by the structural hydroxyls and the different concentrations of transition metals (Fe, Ti and Mg) in biotite and muscovite micas. The estimated activation energies, i.e., 0.33–0.83 eV for biotite and 0.69–1.92 eV for muscovite, were attributed to proton and small polaron conduction, due to the bound water and different oxidation states of Fe.

## 1. Introduction

Micas are complex hydrous aluminum silicate minerals with a layered structure that occur in sedimentary, igneous and metamorphic rocks [1]. From a scientific point of view, mica-bearing rocks are of geophysical importance as the structural anisotropy of their constituents provides constraints on the temperature, conductivity and seismic profiles of Earth’s mantle and transition zone [2,3,4,5]. From a practical perspective, micas are long-outstanding materials with a variety of industrial applications, due to their unique physicochemical, thermal, optical, mechanical, and electrical properties that cannot be found in other natural materials [6,7,8,9]. To name just a few of these properties, micas have excellent thermal stability at high temperatures, high resistance to chemical and environmental corrosion, and high tensile strength, while they possess high electrical resistance and dielectric strength. Because of these superior dielectric characteristics, the electrical and dielectric properties of micas have been extensively investigated in the past few decades, although many of them focused mainly on high electric field measurements [10,11,12,13,14,15,16,17,18,19,20,21,22,23,24,25]. The interest is still undiminished due to the modified structural and dielectric properties of micas upon irradiation and the fact that they combine high working temperature and flexibility as dielectric materials, making them potential candidates for high-temperature energy storage applications [26,27,28,29]. Other peculiar applications concern the use of mica as a potential gate dielectric in organic field-effect transistors (OFETs), as a substrate for biological samples preparation for high resolution microscopy and in the assembly of macroscopic biomimetic polymeric mica films [30,31,32,33].

In the mica group, the dioctahedral muscovite with the general chemical formula KAl_2_(Si_3_Al)O_10_(OH,F)_2_ and the trioctahedral biotite (K(Mg, Fe^2+^)_3_(Al,Fe^3+^)Si_3_O_10_(OH,F)_2_) are the most common hydrous phyllosilicate minerals. Pioneering studies on the electrical properties of muscovite and biotite micas have been limited to measuring the direct current (dc) electrical conductivity and the effect of various factors such as chemical composition, electric field and temperature [11,12,13,18]. Davinson and Yoffe [12] studied the electrical properties of natural and synthetic micas, including ruby muscovite and biotite, in the temperature range 4 to 520 K. They reported that the electrical conductivity is frequency-dependent and is related to the iron content, via electron hopping between ferrous and ferric states. Meunier et al. [13] found that the dc-conductivity of biotite micas increases exponentially with increasing Fe content and suggested that the responsible conduction mechanism is the hopping of small polarons between Fe sites in the crystal.

The necessity of performing electrical measurements in minerals over a broad frequency and temperature range has been consolidated in recent decades, in order to properly identify the electrical conduction mechanisms [34,35]. The frequency dependence of the dielectric properties of different micas has been studied in the past by Chaundry and his coworkers [14,15,16,17,20,21]. In their electrical measurements on muscovite and phlogopite micas at room temperature or below it, they mainly investigated the effects of relative humidity and electrode material, as well as the influence of the structural anisotropy by conducting measurements along and perpendicular to the cleavage planes. The observed loss peak around 1 kHz was related to the hopping of impurity carriers between adjacent sites [17,20]. The effect of high temperature to the dielectric properties of a ruby mica sample perpendicular to the cleavage planes was investigated by Chaundry and Jonscher, in the frequency range 10^−2^–10^4^ Hz and at temperatures up to 956 K [15]. Low-frequency dispersion was observed even at temperatures as high as 900 K and was attributed to the migration of the K^+^ ions in the interlaminar space, with activation energy of 2 eV. Quite recently, Kaur et al. [24,25] measured the dielectric and electrical properties of muscovite and phlogopite micas in the frequency range 0.1 Hz–10 MHz over the temperature range 653 K–873 K. They estimated the activation energies of the conduction process directly from the dc plateaus of the ac-conductivity spectra, giving diverging results, i.e., 0.05 eV for phlogopite and 1.22 eV for muscovite mica, without, however, providing further information on the related conduction mechanisms.

The effect of high temperature on the electrical properties of micas is directly related to dehydration/dehydroxylation processes and the possible associated structural transformations, due to the existence in this type of phyllosilicates of structural water in the form of hydroxyls. The aforementioned processes occurring in micas at elevated temperatures have been investigated over the past years [11,36,37,38,39,40,41]. Ashida et al. [11] reported that the dehydroxylation of muscovite starts above 673 K by the diffusion of water molecules formed from structural hydroxyl ions, without any lattice destruction. Litovchenko and Mazykin [36] attributed the anomalous behavior of the measured dc-conductivity of muscovite between 800 and 1100 K to the dehydroxylation process, caused by protons diffusion to neighboring hydroxyl ions, resulting in water formation. The proton activation energy of 2.1 eV was assigned to this process. Thermal analysis studies of muscovite carried out by Guggenheim et al. [37] showed that the dehydroxylation process occurs over a wide temperature range (748 to 1223 K), suggesting an inhomogeneous process governed by dynamic mechanisms.

In the present work, we investigate the electrical behavior of muscovite and biotite micas measured perpendicular to their cleavage planes, utilizing the powerful technique of complex impedance spectroscopy over wide frequency and temperature ranges that have not been measured so far. Different representations of experimental data, namely impedance Cole-Cole plots, complex electrical conductivity and electric modulus, and modelling of mica samples with properly selected equivalent circuits have been used to distinguish the contributions of the grains interior and grain boundaries, to investigate the conduction mechanisms, and to estimate the corresponding thermal activation energies. In addition, the electrical signatures of the dehydration/dehydroxylation processes of both mica samples occurring at elevated temperatures were identified and presented comparatively.

## 2. Materials and Methods

### 2.1. Experimental Setup

Energy dispersive X-ray fluorescence spectroscopy (S2 Ranger XRF spectrometer, Bruker, Karlsruhe, Germany) was used to determine the concentrations of the major constituent elements of the natural mica samples. The XRF measurements were carried out on pulverized samples at 40 kV by using an Al filter (500 μm) for the heavier elements (Fe, Mn, Ti, Ca, K) and at 20 kV for the lighter elements (Si, Al, Mg, Na). The chemical composition of each mica sample is shown in Table 1.

The complex electrical conductivity measurements were performed by the high-resolution Alpha-N impedance analyzer supplied by Novocontrol [42,43]. In the measured frequency range (10^−2^–10^6^ Hz), the accuracy of impedance measurements (10^3^–10^11^ Ω) is better than 0.1%. The ProboStat high temperature cell (NorECs) combined with a Eurotherm temperature controller was used for performing measurements at elevated temperatures with accuracy of ±1 °C [42]. All measurements were carried out under vacuum obtained by a rotary pump (P ≈ 10^−3^ mbar). Electromagnetic shielding was applied to the high-temperature electrical cell to diminish the electrical noise. Dedicated software (WinDeta and WinFit) was used for data acquisition and analysis in equivalent circuits.

The standard two-electrodes configuration was used in the sandwich arrangement of Tungsten electrodes and disk-shaped mica specimens with a diameter of 20 mm and thicknesses of 250–400 μm. Isothermal impedance spectra were recorded in steps of 40 K or 50 K, during heating and subsequent cooling, over the temperature range 473 K–1173 K. Prior to the electrical measurements in the heating-cooling cycle, each sample was kept for a sufficiently long time at 473 K inside the cell to remove free water and to achieve equilibrium conditions. The latter was verified by taking consecutive measurements without observing any change in the recorded spectra.

### 2.2. Data Representation and Modelling of Samples

For the geometry of the mica samples placed between the round plate electrodes forming a capacitor, a simple equivalent circuit consists of an ohmic resistor in parallel connection to a capacitor. The resistance *R*(*ω*) and the capacitance *C(ω*) are the output values of the impedance analyzer and the total impedance $Z^{*}$ of the sample, which is a complex quantity and is given by the following relation [34]:

(1) $$\frac{1}{Z^{*}\left(\omega \right)}=\frac{1}{R\left(\omega \right)}+i\omega C\left(\omega \right)$$

where ω denotes the angular frequency and $i=\sqrt{-1}$. This equation is represented by a semicircle in the Cole-Cole plot, that is, the diagram of the imaginary *Z*″ versus the real part *Z*′ of impedance. The complex electrical conductivity $\sigma^{*}$ is calculated through the relation:

(2) $$\sigma^{*}\left(\omega \right)=\sigma^{\prime }-i\sigma^{\prime \prime }=\frac{d}{\pi r^{2}R\left(\omega \right)}-i\omega \varepsilon_{o}\left(1-\frac{C\left(\omega \right)}{C_{o}}\right)$$

where $C_{o}=\varepsilon_{ο}\pi r^{2}/d$ is the capacitance of the empty capacitor with electrode spacing *d* and radius *r*, and $\varepsilon_{ο}$ is the permittivity of the vacuum.

An alternative representation of complex impedance data is the electric modulus $M^{*}$ defined as the reciprocal of dielectric permittivity $\left(\varepsilon^{*}=-i/\omega C_{0}Z^{*}\right)$, according to the following expression:

(3) $$M^{*}\left(\omega \right)=\frac{1}{\varepsilon^{*}\left(\omega \right)}={Μ}^{\prime }\left(\omega \right)+iM^{\prime \prime }\left(\omega \right)=\frac{\varepsilon ’}{\varepsilon {’}^{2}+\varepsilon ’{’}^{2}}+i\frac{\varepsilon ’’}{\varepsilon {’}^{2}+\varepsilon ’{’}^{2}}=M_{\infty }\left[1-\overset{\infty }{\underset{0}{\int }}e^{-i\omega t}\left(\frac{d\Phi \left(t\right)}{dt}\right)dt\right]$$

where the function Φ(*t*) defines the attenuation of the electric field within the material, and $M_{\infty }$ equals to 1/*ε*′ at very high frequencies [44]. In the above Equations (2) and (3), the primed and the double-primed quantities correspond to the real and the imaginary part of the complex electrical conductivity *σ*, electric modulus *M* and dielectric permittivity *ε*.

Although each of the aforementioned electrical quantities carries the same information, the electric modulus is preferable to be used in cases where interfacial polarization predominates at low frequencies [45,46]. The $M^{*}$ formalism is appropriate to describe conduction processes, as they appear in the form of loss peaks in the low-frequency range, where the imaginary part of the dielectric permittivity increases steadily, overlapping any other contribution [42,43].

In the case of crystalline materials such as minerals, the simple approach of R-C elements in parallel is not appropriate to describe their electrical response to an alternating electric field, as a considerable depression of the ideal semicircle is usually observed in the Cole-Cole plots [34,47,48]. A common empirical model describes this deviation by substituting the ideal capacitor in the R-C equivalent circuit with a constant phase element (CPE) [34]. The advantage of the R-CPE equivalent circuit is that the real part of ac-conductivity and thus the dc-conductivity ($\sigma_{dc}$) can still be derived by fitting the resistance *R*, according to Equation (2). However, the physical interpretation of the CPE is doubtful, but in cases of crystalline materials, it has been related to the inhomogeneous distribution of defects around the grain boundaries [49].

The electrical impedance of a CPE is expressed through the following definition:

(4) $$Z_{CPE}=\frac{1}{Q{\left(i\omega \right)}^{n}}=\frac{1}{Q\omega^{n}}\left[cos\left(-n\frac{\pi }{2}\right)+isin\left(-n\frac{\pi }{2}\right)\right]$$

where Q is the pre-factor of the CPE and *n* its exponent. By substituting the capacitor impedance in Equation (1) with the impedance of the CPE (refer to Equation (4)), we get the following expression for the total impedance of the R-CPE elements in parallel connection [42]:

(5) $$Z\left(\omega \right)=Z^{’}+iZ^{\prime \prime }=\left[R+QR^{2}\omega^{n}\cos\left(n\frac{\pi }{2}\right)\right]/D-i\left[QR^{2}\omega^{n}\sin\left(n\frac{\pi }{2}\right)\right]/D$$

where $D=1+2QR\omega^{n}\cos\left(n\frac{\pi }{2}\right)+Q^{2}R^{2}\omega^{2n}$.

In the present case, a combination of two or three R-CPE circuits in series were used to model the electrical impedance of the measured mica samples, while the electric modulus formalism was used to identify the related conduction mechanisms and the dehydration/dehydroxylation processes during the heating of the samples at elevated temperatures.

## 3. Experimental Results and Analysis

**Figure 1:**
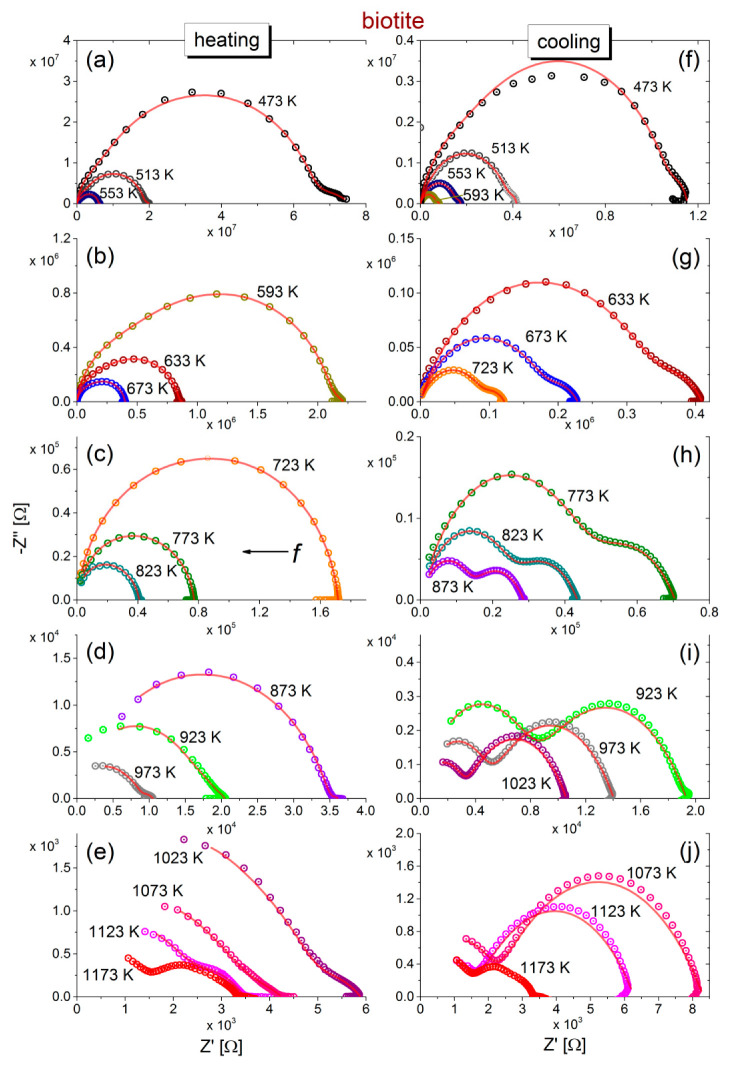
Cole-Cole plots of impedance (−Z″ vs. Z′) during the heating from 473 to 1173 K (**a**–**e**) and subsequent cooling (**f**–**j**) to 473 K of biotite mica sample. Experimental data are shown with open circles. Frequency increases from right (10^−2^ Hz) to left (10^6^) Hz. The solid (red) lines correspond to fitting with an equivalent circuit. Note the different scales that are used in each plot of the heating-cooling cycle.

**Figure 2:**
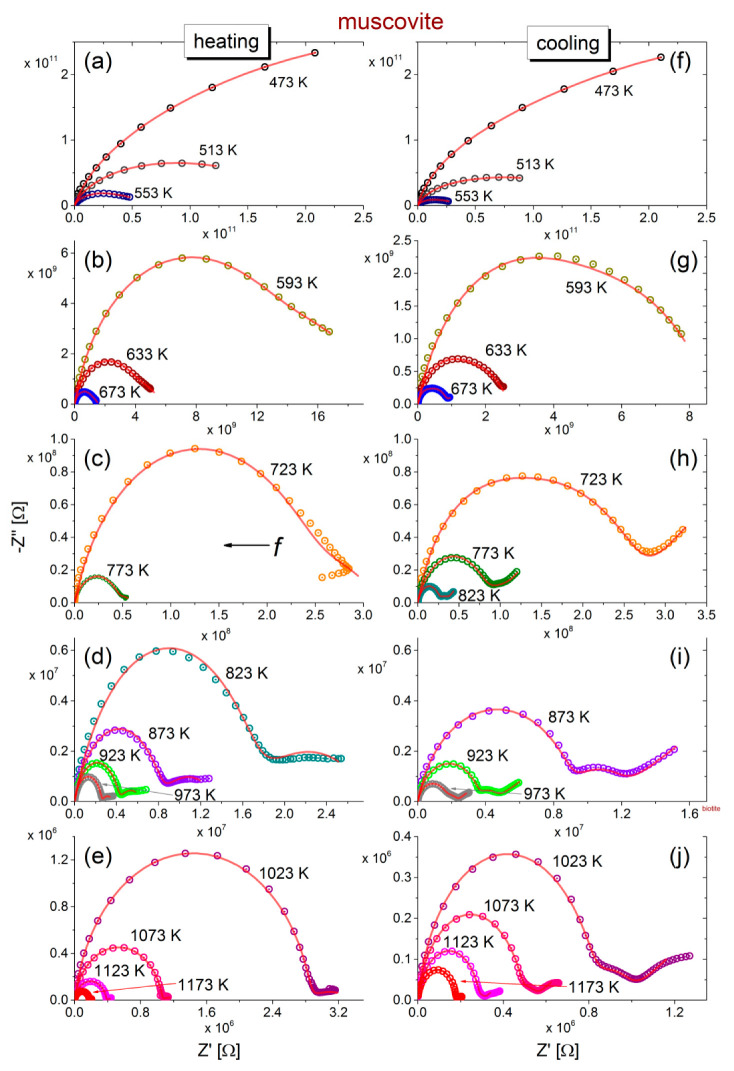
Cole-Cole plots of impedance (−Z″ vs. Z′) during the heating (**a**–**e**) and subsequent cooling (**f**–**j**) of muscovite mica samples. Experimental data are shown with open circles. Frequency increases from right (10^−2^ Hz) to left (10^6^) Hz. The solid lines correspond to fitting with equivalent circuits of R-CPE elements in series. Note that different scales have been used in each plot during the heating-cooling cycle.

The negative imaginary part −Z″ versus the real part Z′ of complex impedance (Cole-Cole plot) during the heating of biotite and muscovite samples to 1173 K and their subsequent cooling to 473 K is shown in Figure 1a–j and Figure 2a–j. During heating of biotite to 473 K (refer to Figure 1a), the Cole-Cole plot consists of a single depressed semicircle accompanied by an additional feature in the low frequency range, which, however, becomes less intense as the temperature increases. Incomplete semicircles are recorded at temperatures higher than 873 K, while an additional feature is also observed at low frequencies, above 923 K. Upon subsequent cooling of biotite, two overlapping semicircles are observed at low and higher frequencies, the size of which varies inversely with each other. That is, the radius of the low-frequency semicircle decreases, while that of the high frequency semicircle increases, as temperature decreases. In general, impedance varies by more than 4 orders of magnitude in the measured temperature range during heating, and the recorded spectra at the same temperature during gradual cooling exhibit different characteristics.

Regarding the muscovite sample (Figure 2), incomplete semicircles are initially observed during heating which, as temperature increases above 773 K, are evolved into complete ones, accompanied by an additional feature at low frequencies. The latter is more pronounced during the subsequent cooling (refer to Figure 2h–j) and comprises a depressed semicircle and a tail. This tail is attributed to unwanted electrode polarization effects, which are often observed at low frequencies.

The impedance values decrease by almost 5 orders of magnitude with increasing temperature, but the shape of the curves exhibit similar features in each part of the heating-cooling cycle.

In a first approximation, by neglecting the contribution of the low-frequency features and any deviation of the experimental data from the semicircular shape, a single R-CPE equivalent circuit could be roughly used to model the mica samples and derive their total (bulk) conductivity as a function of temperature. Oversimplified approaches as the aforementioned one and even the simpler R-C circuit in parallel have often been used to estimate the bulk conductivity of minerals [50,51,52]. However, as the recorded impedance spectra of the mica samples exhibit more complicated spectral features, we are obliged to analyze them in more detail, in order to separate different contributions to the overall bulk conductivity.

In general, the observed sequence of a depressed high-frequency semicircle followed by a second one and a tail at low frequencies is representative of the electrical behavior of polycrystalline minerals measured over a wide range of frequencies [34]. The main semicircle observed at high frequencies is attributed to the intrinsic conductivity of the grains, while the next one at the medium frequency range is due to the contribution of the grain boundaries. A tail usually observed at low frequencies (below a few Hz) is due to the unwanted effects of electrodes polarization, caused by the accumulation of charges in the electrode-specimen interface. In the case of the mica samples, the grains are actually the aluminosilicate sheets arranged in the laminate structure. The boundaries of these sheets may also form a conducting pathway acting in series with the packed sheets.

Based on the previous observations and considerations, the total impedance of the mica samples can be considered as the sum of three different contributions, as follows:

(6) $$Z_{tot}=Z_{R_{\mathrm{gi}}\|{\mathrm{CPE}}_{\mathrm{gi}}}+Z_{R_{\mathrm{gb}}\|{\mathrm{CPE}}_{\mathrm{gb}}}+Z_{R_{\mathrm{ep}}\|{\mathrm{CPE}}_{\mathrm{ep}}}$$

where $Z_{R\|\mathrm{CPE}}$ indicates the impedance of the circuit consisting of the resistor R in parallel to the CPE, and the subscripts gi, gb and ep stand for grains interior, grain boundaries and electrodes polarization, respectively. At certain measured temperature ranges of biotite and muscovite samples, the last term in Equation (6) may be omitted, as there is no considerable indication of electrode effects in the measured frequency range. Consequently, according to Equation (5), the real and the imaginary part of the total impedance $Z_{tot}$ are given as follows:

(7) $$Z^{\prime }=\underset{j}{\sum }\frac{R_{j}+Q_{j}R_{j}^{2}\omega^{n_{j}}\cos\left(n_{j}\frac{\pi }{2}\right)}{1+2Q_{j}R_{j}\omega^{n_{j}}\cos\left(n_{j}\frac{\pi }{2}\right)+Q_{j}^{2}R_{j}^{2}\omega^{2n_{j}}}$$

and

(8) $$Z^{\prime \prime }=-\underset{j}{\sum }\frac{Q_{j}R_{j}^{2}\omega^{n_{j}}\sin\left(n_{j}\frac{\pi }{2}\right)}{1+2Q_{j}R_{j}\omega^{n_{j}}\cos\left(n_{j}\frac{\pi }{2}\right)+Q_{j}^{2}R_{j}^{2}\omega^{2n_{j}}}$$

where the sum in each equation includes the contribution of grains interior, grain boundaries and that of electrode effects where applicable.

**Figure 3:**
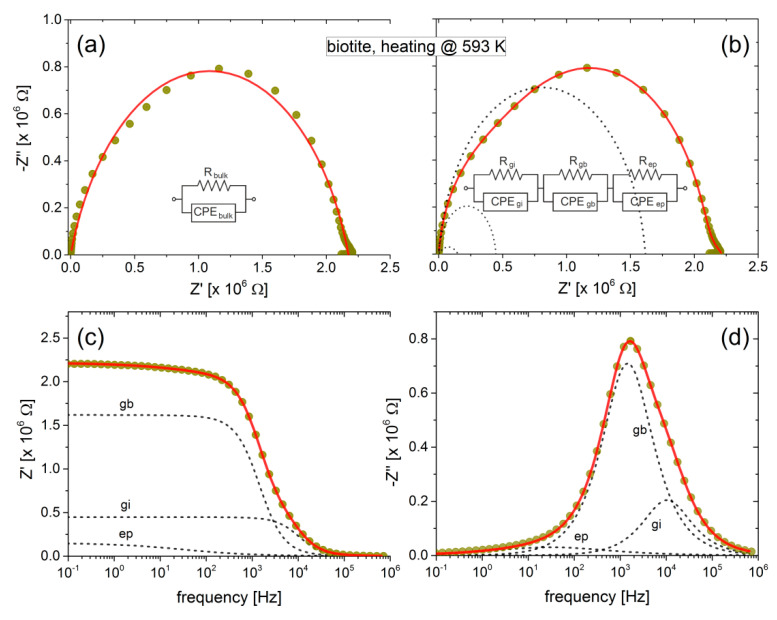
Cole-Cole plot of biotite at 593 K during the heating procedure (refer to Figure 1b). In (**a**), the solid (red) line corresponds to the fitting of data with a resistor (R_bulk_) and a constant phase element (CPE_bulk_) in parallel. In (**b**), three R-CPE circuit elements in series have been used to fit the same experimental data. The three dashed semicircles correspond to each one of the R-CPE circuits. In (**c**,**d**), the real and imaginary part of impedance *Z* has been plotted as a function of frequency. Dashed lines correspond to the contribution of each R-CPE circuit.

The feasibility of the above analysis is clearly demonstrated in Figure 3, where the complex impedance of biotite sample heated at 593 K is shown in the Cole-Cole representation and as a function of frequency. Although the bulk conductivity can be estimated satisfactory considering a single R-CPE circuit (or even a R-C circuit) in parallel, the combination of additional elements is inevitable for the accurate fitting that will reveal the existence of possible conduction mechanisms. Indeed, according to Figure 3a,b, the bulk conductivity of the biotite sample is about the same (≈2.2 MΩ), regardless of the use of one or three R-CPE circuits. However, in the latter case, the more complicated modelling of the sample may distinguish the contribution of grains interior and grain boundaries to the overall conductivity of the sample.

**Figure 4:**
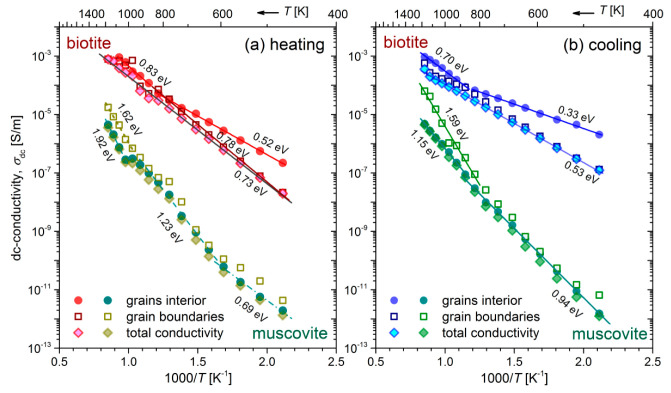
Arrhenius plots of dc-conductivity during the heating-cooling cycle of biotite and muscovite mica samples, during (**a**) gradual heating to 1173 K and (**b**) subsequent cooling to 473 K. The thermal activation energies *E_a_* are denoted for each linear region of the plots (refer also to Table 2). For their explanation, see the text.

The fittings of the equivalent circuits for biotite and muscovite samples to the experimental data according to Equations (7) and (8) are shown in Figure 1 and Figure 2 with solid (red) lines, at all measured temperatures, during the heating-cooling cycles. Visual inspection of the fitting curves shows good correlation with the experimental data. Based on the fitting values of resistance and taking into account the geometrical factor (refer to the real part of Equation (2)), the dc-conductivity ($\sigma_{dc}$) values of both mica samples have been calculated in the measured temperature range of the heating-cooling cycle (473–1173 K). The logarithm of the calculated dc-conductivity attributed to grains interior and grain boundaries of both mica samples during the heating-cooling cycle, as a function of the inverse temperature (Arrhenius plot), is shown in Figure 4. The total dc-conductivity of each sample has been also included in Figure 4 for comparison. We observe that during heating of the biotite sample, the total $\sigma_{dc}$ increases by almost 5 orders of magnitude in the measured temperature range, reaching the value ~10^−3^ S/m at 1173 K. After the subsequent cooling to the initial temperature (473 K), $\sigma_{dc}$ remains roughly 5 times higher than its value before heating. As for the muscovite sample, the total conductivity is 2–5 orders of magnitude lower than that of biotite, depending on the measured temperature and the direction of cycle, i.e., if the measurements have been carried out during the heating or the cooling procedure. Furthermore, a remarkable variation of 7 orders of magnitude is observed in $\sigma_{dc}$, either during heating at 1173 K or subsequent cooling to 473 K. In contrast to the biotite sample, the variation of $\sigma_{dc}$ in muscovite during heating is rather more complicated, making it difficult to distinguish linear regions, even in narrow temperature ranges (refer to Figure 4a). However, this behavior becomes more distinct during the cooling, where two linear regions are clearly observed. However, in both mica samples during heating above ~800 K, fluctuations of $\sigma_{dc}$ are more pronounced.

It is worth mentioning that the total dc-conductivity of biotite exhibits a rather simplified or even misleading behavior, as it varies linearly over the entire measured temperature range, either during heating or cooling, indicating in this way a single conduction mechanism (refer to Figure 4b). Thus, as we proposed previously, the contribution of both grains interior and grain boundaries to the electrical conductivity should be examined separately, for the proper identification of the related conduction mechanisms in both mica samples.

A linear variation of the electrical dc-conductivity $\sigma_{dc}$ in the Arrhenius plot (refer to Figure 4) suggests a single conduction mechanism, according to the relation:

(9) $$\sigma_{dc}\left(T\right)=\sigma_{o}exp\left(-E_{a}/k_{B}T\right)$$

where $\sigma_{o}$ is the pre-exponential factor, $E_{a}$ is the energy of the thermally activated conduction process and $k_{B}$ is Boltzmann’s constant [34]. The thermal activation energy $E_{a}$ is determined directly from the slope of each of the linear regions shown in Figure 4. These calculated values are summarized in Table 2, for both mica samples and the different contributions (gi and gb).

A general finding is that the activation energies of biotite sample corresponding to grains interior, and grain boundaries are lower in the cooling process than those during heating. Indeed, the *E*_a_ of the two branches related to the grains interior, i.e., the values 0.52 eV and 0.83 eV (refer to Figure 4a) decrease to 0.33 eV and 0.70 eV, respectively, during the cooling process. Similarly, the activation energy due to grain boundaries decreases from 0.78 eV to around 0.53 eV during cooling. This behavior also holds to some extent for the muscovite sample, as concerns the medium and high temperature ranges. In this case, higher *E*_a_ have been derived. The fact that the gradual cooling of mica samples is not a reversible process in terms of $\sigma_{dc}$ variation suggests that a transformation takes place during the heating procedure. The latter conclusion, as well as the associated conduction mechanisms will be discussed in conjunction with the following results in the next section.

**Figure 5:**
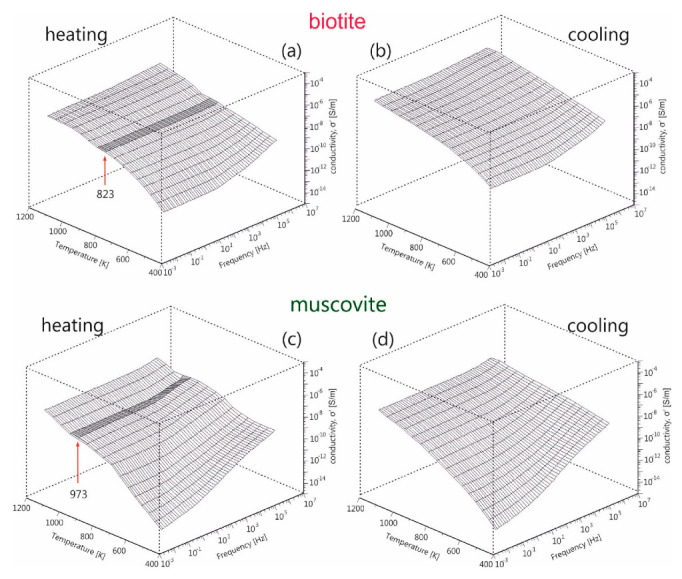
3D-plane plots of real part of electrical ac-conductivity (σ′) as a function of frequency and temperature up to 1173K, for (**a**,**b**) biotite and (**c**,**d**) muscovite mica for both heating and cooling procedures. The dark areas around 823 K and 973 K for biotite and muscovite mica indicate the discontinuity of the conductivity spectra, which may be related to the dehydroxylation process.

In order to explore further the electrical behavior of the mica samples, in addition to the dc-conductivity, the frequency dependence of electrical conductivity has been examined. The real part of ac-conductivity, *σ*′, during heating and subsequent cooling of biotite and muscovite mica samples is depicted in Figure 5a–d, over the entire frequency and temperature measured ranges. Dispersion is observed in both samples, either during heating or cooling, especially in the low temperatures region. It is noteworthy that the conductivity is constantly decreasing as frequency decreases, which is more pronounced at lower temperatures. This makes it impossible to determine in accuracy the dc-plateau in each recorded spectrum and the corresponding dc-conductivity value. Thus, the estimation of $\sigma_{dc}$ values through modelling with equivalent circuits as previously presented is inevitable, which is in opposition to the direct estimation from the ac-conductivity spectra, reported by Kaur et al. [25].

A peculiar behavior is observed during heating of both samples (denoted with dark grey in Figure 5a,c), which appears as a discontinuity in conductivity at 823 K for biotite and at 973 K for muscovite. A conduction relaxation feature i.e., a non-uniform fluctuation of ac-conductivity, as temperature increases up to 973 K is also observed during heating of muscovite. The above features disappear during the subsequent cooling of samples (refer to Figure 5b,d). These features should be related to the dehydration/dehydroxylation processes taking place during the gradual heating of the mica samples.

**Figure 6:**
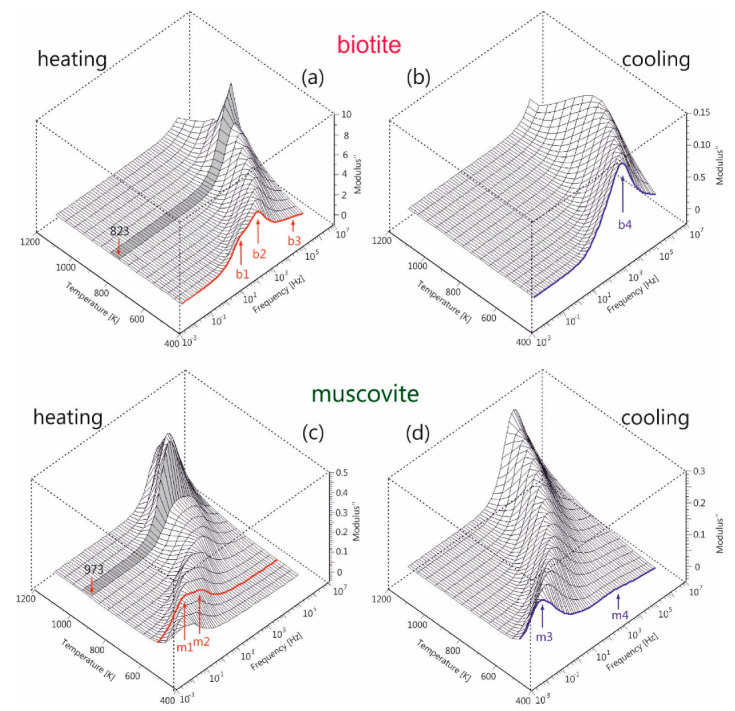
3D-plane plots of imaginary part of electric modulus (*M*″) as a function of frequency and temperature for biotite (**a**,**b**) and muscovite (**c**,**d**) mica, during gradual heating at 1173 K and subsequent cooling to 473 K. See text for details.

The alternative approach of the electric modulus M* can emphasize the abovementioned behavior of ac-conductivity, as the electrode effects are depressed and different conduction mechanisms appear as relaxation peaks in this type of data representation. The imaginary part of the electric modulus (M″) of both mica samples during the heating-cooling cycles is depicted in Figure 6, as 3D-plane plots, for the measured frequency and temperature ranges. During heating of biotite, two overlapped relaxation peaks (denoted as b1, b2 in Figure 6a) are observed in the medium frequency range, which are shifted at higher frequencies as the temperature increases. An additional feature (b3) with low intensity also appears at higher frequencies but it shifts out of the measured frequency range with increasing temperature. An abrupt increase in intensity of the main peak is observed after 823 K. During the subsequent cooling, the observed broad peak (b4) lies within the measured frequency range at temperatures below 773 K, while its intensity remains constant.

A similar situation is also observed during the heating of muscovite. A combination of two peaks (denoted as m1 and m2 in Figure 6c) appears initially in the low frequency region. These peaks evolve into a broader feature, whose intensity increases abruptly after 973 K and then gradually decreases again. During the subsequent cooling, a broad intense peak (m3) is observed initially at high frequencies, which shifts to lower frequencies and whose intensity gradually decreases as the temperature decreases. At the same time, a new weak peak (m4) emerges at temperatures below 873 K, following the evolution of the m3 peak.

It should be noted that the appearance of the sharp increase of peak intensity at 873 K for biotite (Figure 6a) and at 1023 K for muscovite (Figure 6c) during their heating coincides with the observed discontinuity of ac-conductivity at the same temperatures (refer to Figure 5a,c). This implies that the underlying phenomenon, which is more pronounced in the electric modulus representation but is absent during cooling, should be related to the dehydration/dehydroxylation of both samples occurring at different temperatures. These findings are consistent with those of Mazzucato et al. [41], who studied the dehydroxylation process of muscovite by x-rays powder diffraction and stated that the reaction takes place in the range 973 K–1273 K. Reported thermal analysis studies of muscovite suggested that the dehydroxylation process occurs in a narrower temperature range, 1073 K to 1173K [53]. The subsequent study of muscovite by x-rays and neutron diffraction at high-T, carried out by Gridi-Bennadji et al. [38], suggested that microstructural transformations in the silicate layers take place but the layered structure is maintained up to 1368 K.

As a consequence of the processes occurring during gradual heating, *M*″ exhibits a complicated behavior, where the conduction relaxation peaks are affected by the water-induced spectral changes. However, during gradual cooling, a uniform evolution of the peaks is observed, without any discontinuities in the successive recorded spectra. Thus, the additional analysis of the modulus spectra during cooling could give insights to the intrinsic conduction mechanisms.

**Figure 7:**
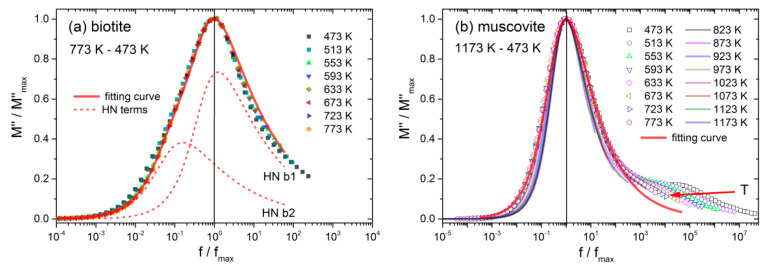
The normalized imaginary part of electric modulus (*M*″/*M*″_max_) as a function of normalized frequency (*f*/*f_max_*) during the cooling to 473 K for (**a**) biotite and (**b**) muscovite micas. Solid red lines correspond to fitting of data with the Havriliak-Negami relaxation function of Equation (10).

The normalized plots of *M*″ versus the frequency of both mica samples during the cooling procedure are depicted in Figure 7a,b. These master curves reveal whether the shape of the spectra remains unchanged as the temperature changes. The validity of the so-called time-temperature superposition principle (TTSP) indicates that the related conduction mechanisms remain the same at a certain temperature range [54,55]. Indeed, according to Figure 7a, during gradual cooling of biotite, the conductivity relaxation peak (b4), which is observed below 773 K, maintains its shape up to 473 K. The latter implies that the related conduction mechanisms of the dynamic process are independent of temperature, at least in the temperature range 473 K–773 K.

The underlying conduction mechanisms related to the b4 relaxation peak in the limited temperature range, 473 K–773 K, can be revealed by fitting the experimental data to a Havriliak-Negami (HN) relaxation function, according to the following expression:

(10) $$M^{\prime \prime }\left(\omega \right)=\overset{2}{\underset{j=1}{\sum }}\left[\frac{\Delta {Μ}_{j}}{{\left(1+{\left(i\omega \tau_{{Μ}_{j}^{*}}\right)}^{\alpha_{j}}\right)}^{\beta_{j}}}+M_{\infty j}\right]$$

where *ΔM* is the relaxation strength, $\tau_{M^{*}}$ is a characteristic relaxation time and $M_{\infty }$ corresponds to the value of *M*′ at infinite frequency [56]. The constants *α* and *β* determine the shape of the peak, i.e., the broadness and its asymmetry, respectively. Two HN terms are necessary to describe accurately the master curve of biotite during the gradual cooling process. These two terms (viz, HN-b1, HN-b2) are shown with dashed red lines in Figure 7a, and the fitting parameters are summarized in Table 3.

Regarding the muscovite sample during the gradual cooling (Figure 7b), the master plot of *M*″ incorporates two distinct behaviors described by the peaks m3 and m4 (refer to Figure 6d). The weak peak (m4) is shifted towards lower frequencies with increasing temperature, implying that the TTSP does not hold in this case. On the other hand, the main relaxation peak (m3) retains its spectral shape from 473 K up to 773 K, but at temperatures higher than 823 K, the full-width at half maximum (FWHM) reduces gradually, resulting in a sharp peak at 1173 K. It is evident that the latter behavior should be related to the gradual dehydration of the sample. A single HN relaxation term is sufficient to fit the master curve in the range 473 K–773 K, as it is depicted in Figure 7b. The corresponding fitting parameters are included in Table 3. At temperatures higher than 773 K, successive fittings with HN functions are necessary to describe the dynamic effect of temperature.

**Figure 8:**
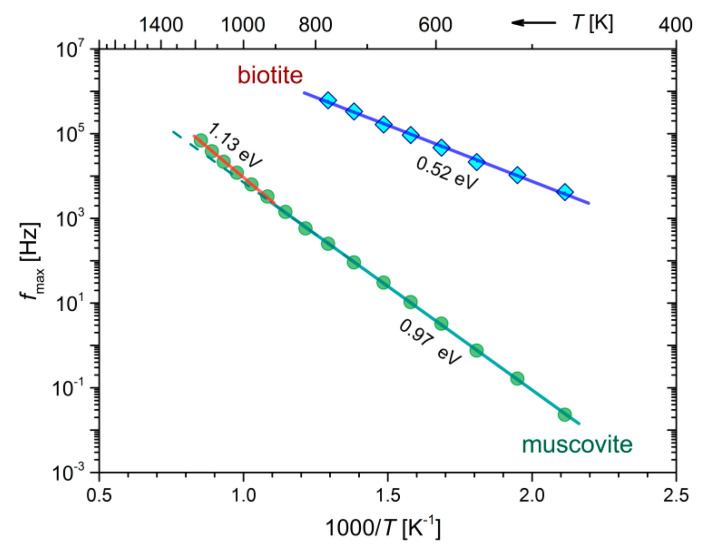
Arrhenius plot of the peak frequencies, *f*_max_ in the *M*″ spectra of mica samples, during their gradual cooling. The activation energies are shown in each linear region.

In the previous temperature regions where the peaks appear in the *M*″ spectra of both samples during cooling (see Figure 7a,b), the corresponding peak frequencies, *f*_max_, are shown in the form of Arrhenius plots in Figure 8. The calculated activation energies corresponding to each lineal region of *f*_max_ in Figure 8 are in fact the same as those of the dc-conductivity during the cooling of biotite and muscovite (refer to Figure 4b and Table 2). This finding suggests that identical conduction mechanisms describe both *f*_max_ and $\sigma_{dc}$ variations in the measured temperature range.

Recalling that the HN relaxation function corresponds to a distribution of relaxation times *g*(*τ*) [56], the relaxation time distribution *G*(*τ*) of the superposition of the HN terms as in Equation (10), is given by the following expression:

(11) $$G\left(\tau \right)=\underset{j}{\sum }\Delta M_{j}g_{j}\left(\tau \right)/\underset{j}{\sum }\Delta M_{j}$$

**Figure 9:**
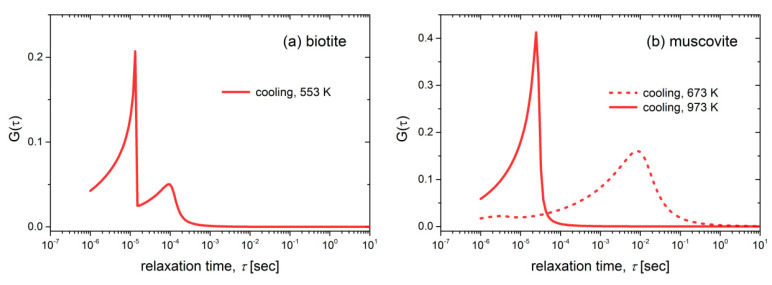
Distributions of the relaxation times G(τ) for (**a**) biotite and (**b**) muscovite samples at selected temperatures during their cooling.

In the above equation, the distribution of relaxation times $g_{j}\left(\tau \right)$ of the *j*th HN term has been expressed by an analytical expression by Havriliak and Negami, where the parameters of Equation (10) are also included [56,57]. The above transformation of the *M*″ master curves of Figure 7a,b leads to the distributions of the relaxation times *G*(*τ*) depicted in Figure 9a,b. It should be noted that these distributions were not normalized with respect to frequency in order to correspond to the real values of relaxation times. Two neighboring fast distributions are observed during the cooling of biotite at 553 K, i.e., a small one around 10^−4^ s followed by a sharp and faster process with a cut-off frequency at around 10^−5^ s. Regarding the muscovite sample, a broad distribution is observed at 673 K, which corresponds to a slow process (10^−2^ s). This is related to a single conduction mechanism but with a wide distribution of relaxation times. At higher temperatures (973 K), a sharp distribution of a fast process is observed with a cut-off frequency of ~2 × 10^−4^ s.

## 4. Discussion

Although the electrical properties of micas have been studied extensively over the years, the potential of complex impedance spectroscopy has not been explored in detail in this type of aluminum silicate minerals or even in other types of minerals and rocks. According to our detailed analysis, the complicated electrical response of the mica samples at elevated temperatures necessitates the separation of the different contributions, that is, the conductivity of the grains interior and the grain boundaries, as was the case of other minerals and rocks [58,59,60]. On the other hand, the representation of the experimental data with different formalisms was necessary to extract complementary information about the conduction mechanisms and the dehydration processes that took place during the gradual heating of mica samples.

Specifically, the Cole-Cole plots of impedance distinguished the different contributions to the overall electrical conductivity of biotite and muscovite micas, making it feasible to calculate the thermal activation energies of grains interior and grain boundaries (refer to Figure 4). The *E*_a_ due to the grains interior is 0.52 eV for biotite and temperature-dependent for muscovite (0.69 eV–1.23 eV) during their heating at the temperature range 473 K–823 K, while it decreases to 0.33 eV and 0.94 eV for biotite and muscovite, respectively, during their gradual cooling. Such low values of *E*_a_ have been reported by Rüscher and Gall [22] for iron-bearing trioctahedral phyllosillicates, namely 0.3 eV–0.6 eV for biotite Moen and vermiculite Benahavisin in the temperature range 300 K–900 K. These values were attributed to polaron conduction and disordering of the samples. Meunier et al. [13] also reported low values of *E*_a_ (0.50 eV–0.72 eV) for dc-conductivity measurements of biotite that were associated to hopping of small polarons. Activation energy of 1.22 eV has been reported for muscovite mica by Kaur et al. in the range 653 K–853 K, but without providing any explanation [25]. Chaundry and Jonscher reported a high *E*_a_ of 2 eV for a ruby muscovite sample and they attributed this value to the migration of K ions in the interlayer space [15]. However, this explanation should be ruled out in our case as the amount of potassium is about the same in our samples (refer to Table 1), but different low values of *E*_a_ have been found for each mica sample. It should be noted that a decrease in conductivity with increasing the sodium content has been reported by Guseivov for muscovite and phlogopite micas [61]. This could also explain to some extent the reduced measured conductivity of the muscovite sample with a Na content of 2.2 wt% (refer to Table 1).

Generally, in the case of Fe-bearing hydrous minerals, the electron charge transfer between ferrous and ferric states of structural iron, as well as the diffusion of H (proton) or H-related defects, are the main mechanisms of conduction at moderate temperatures, while the predominant contribution of ionic conduction occurs at high temperatures, i.e., above ~1100 K [35,62,63]. In the first case, the mechanism is better known as “small polaron” conduction, where the hopping of electron with low mobility causes the local distortion of the lattice, and the corresponding *E*_a_ varies in the range 0.7 eV–1.5 eV [35,64,65,66]. Lower values of *E*_a_ such as ~0.8 eV for olivine and 0.6 eV for wadsleyite are consistent with proton conduction due to the high mobility of hydrogen species [35,67].

In the present case, the electrical conductivity of biotite and muscovite micas is affected by the different concentrations of Fe, Ti and Mg transition metals (refer to Table 1), resulting in substantial variations of conductivity between these two samples (refer to Figure 4). The presence of the higher iron concentration (17.1 wt%) in biotite sample with respect to muscovite (5.3 wt%) should play the dominant role to the higher measured values of ac-conductivity in biotite, through the polaron conduction mechanism. Furthermore, the existence of Mg in the biotite sample (7.5 wt%) seems to provide lattice sites for the accommodation of ferrous ions, which contribute to the small polaron conduction and therefore to the increased electrical conductivity [61]. We propose that the small polaron conduction mechanism should take place in biotite and muscovite micas at T > 873 K, where the change in slope is observed in the Arrhenius plots (refer to Figure 4). At temperatures lower than 873 K, the low values of the estimated thermal activation energies *E*_a_, i.e., 0.52 eV–0.73 eV during heating of both samples, suggest that the proton conduction due to the bound water should be the dominant mechanism. The fact that the *E*_a_ of grains interior and grain boundaries of biotite sample is found to be lower during the cooling run as compared to that during heating, while the corresponding *σ*_dc_ is higher, could be explained on the basis of the microstructural changes that take place at high temperatures due to the hydroxylation reaction. According to reported studies [38,41], the latter process extends beyond 1173 K where our measurements were carried out and the rearrangement of the remaining hydroxyls could contribute to the higher conductivity values measured during the cooling run (refer to Figure 4b). In addition, the irreversible redox reactions that take place during heating may change the oxidation state of iron and the content of the structural hydroxyl and oxygen [55].

According to our analysis in terms of the modulus representation, where *E*_a_ of *f*_max_ is the same as that of $\sigma_{dc}$ during cooling (refer to Figure 8), the observed peaks in *M*″ spectra were identified as conduction relaxation peaks associated with the aforementioned conduction mechanisms. The different behavior of *M*″ during the gradual cooling compared to that of the heating cycle (refer to Figure 6) should be attributed to the dehydration and dehydroxylation processes that take place during the heating run. The dehydration of interlayer water is a gradual process that occurs when both mica samples are heated from 473 K at elevated temperatures and affects the evolution of the observed conduction relaxation peaks (b1, b2 and b3 for biotite, m1, m2 for muscovite) in a rather complicated way. At higher temperatures where the bound water should have been desorbed, the contribution of the structural hydroxyls to the formation of water molecules seems to have its own electrical signature, that is, the abrupt change of peak shape and intensity in *M*″ spectra, which is observed in both samples at different temperatures. That is, the dehydroxylation reaction starts at a lower temperature in the case of biotite (~873 K) compared to that of muscovite (~1073 K). During the subsequent cooling of the biotite sample, according to the master plot of *M*″ in the temperature range 773 K–473 K (refer to Figure 7a), a temperature-independent mechanism dominates with two distinct distribution of relaxation times, as it is shown in Figure 9a. This TTSP is evidence of the absence of loosely bound water after the dehydration of the biotite sample and should be related to fast processes of proton conduction in grains or between them with relaxation times 10^−4^–10^−5^ s. In the case of the muscovite sample, the broad distribution of relaxation times observed at the same temperature range as before (773 K–473 K) should be attributed to the same reason. At temperatures higher than 923 K, the sharp relaxation peak with small relaxation times (~10^−5^ s) should be attributed to the small polaron conduction.

## 5. Conclusions

A comparative study of the electrical properties of biotite and muscovite micas from 473 K to 1173 K was presented, utilizing the powerful technique of broadband electrical impedance spectroscopy in the frequency range 10 mHz–1 MHz. Cole-Cole plots of impedance were used to separate the contributions of grains interior and grain boundaries and to estimate the corresponding activation energies in each case. Depending on the measured temperature, biotite mica exhibits 2 to 4 orders of magnitude higher dc-conductivity as compared to muscovite, due to its higher content of Fe and other transition metals (Mg and Ti). The calculated activation energies indicate that, in the measured temperature range, the dominant conduction mechanisms in both micas are identified as proton conduction at T < 823 K (*E*_a_ = 0.33–0.94 eV) and hopping of small polarons at higher temperatures (*E*_a_ = 0.70–1.15 eV). The thorough analysis of experimental data in terms of the electric modulus representation reveals the role of bound water existing in the form of interlayer molecules or as structural hydroxyls. In this type of representation, the electrical signatures of dehydration/dehydroxylation processes are highlighted during the heating-cooling cycle of both mica samples.

## Figures and Tables

**Table 1:** Chemical composition (wt%) of muscovite and biotite samples before measurements.

Mica	SiO_2_	Al_2_O_3_	Fe_2_O_3_^1^	TiO_2_	MgO	K_2_O	Na_2_O	CaO	MnO	LOI ^2^	Total
muscovite	46.1	29.3	5.3	0.2	0.0	9.4	2.2	0.3	0.2	4.42	97.5
biotite	44.9	14.1	17.1	2.9	7.5	10.2	0.0	0.2	0.7	0.84	98.5

^1^ All Fe measured as Fe^3+^, ^2^ Loss on ignition.

**Table 2:** Activation energies (*E*_a_) of Arrhenius type behavior of dc-conductivity (straight lines in Figure 4) during heating and subsequent cooling of biotite and muscovite mica samples. All values are in eV. Error values are derived from the fittings.

	Biotite		Muscovite	
	Heating	Cooling	Heating	Cooling
total	0.73 ± 0.01	0.53 ± 0.01	-	-
grains interior 1	0.52 ± 0.01	0.33 ± 0.01	(0.69–1.23) ± 0.04	0.94 ± 0.01
grains interior 2	0.83 ± 0.06	0.70 ± 0.02	1.92 ± 0.04	1.15 ± 0.02
grain boundaries	0.78 ± 0.01	0.57 ± 0.01	1.62 ± 0.08	1.59 ± 0.10

**Table 3:** Fitting parameters of the (un-normalized) HN relaxation function (Equation (10)) that describes the master curves of biotite and muscovite micas during their gradual cooling (refer to Figure 7a,b).

Mica	HN Peaks	Δ*Μ*	*τ*_Μ_ (s)	*α*	*β*
biotite	HN-b1	0.2936	1.472 × 10^−5^	1	0.3447
	HN-b2	0.1645	1.203 × 10^−4^	0.9023	0.3771
muscovite	HN-m	0.7071	1.334 × 10^−2^	0.7721	0.4635
